# PCTK3/CDK18 regulates cell migration and adhesion by negatively modulating FAK activity

**DOI:** 10.1038/srep45545

**Published:** 2017-03-31

**Authors:** Shinya Matsuda, Kohei Kawamoto, Kenji Miyamoto, Akihiko Tsuji, Keizo Yuasa

**Affiliations:** 1 Department of Biological Science and Technology, Tokushima University Graduate School, Minamijosanjima, Tokushima 770-8506, Japan; 2 Department of Bioscience and Bioindustry, Tokushima University Graduate School, Minamijosanjima, Tokushima 770-8513, Japan

## Abstract

PCTAIRE kinase 3 (PCTK3) is a member of the cyclin dependent kinase family, but its physiological function remains unknown. We previously reported that PCTK3-knockdown HEK293T cells showed actin accumulation at the leading edge, suggesting that PCTK3 is involved in the regulation of actin reorganization. In this study, we investigated the physiological function and downstream signal transduction molecules of PCTK3. PCTK3 knockdown in HEK293T cells increased cell motility and RhoA/Rho-associated kinase activity as compared with control cells. We also found that phosphorylation at residue Tyr-397 in focal adhesion kinase (FAK) was increased in PCTK3-knockdown cells. FAK phosphorylation at Tyr-397 was increased in response to fibronectin stimulation, whereas its phosphorylation was suppressed by PCTK3. In addition, excessive expression of PCTK3 led to the formation of filopodia during the early stages of cell adhesion in HeLa cells. These results indicate that PCTK3 controls actin cytoskeleton dynamics by negatively regulating the FAK/Rho signaling pathway.

The actin cytoskeleton is required for the regulation of various cell events, such as cell migration, adhesion, and cytokinesis. Actin cytoskeleton dynamics are altered in response to extracellular stimulation and cell cycle progression, which provides mechanical strength and contractile force to the cell body^1^. The extension and retraction of actin filaments is regulated by cross-talk between actin binding proteins and the Rho GTPase family, including RhoA, Rac1, and Cdc42^2^. The Rho GTPase family regulates fundamental processes, such as cell movement, polarity, and division in eukaryotic cells^3^. Active GTP-bound Rho GTPase leads to stress fiber assembly and characteristic membrane protrusion and expansion, such as lamellipodia and filopodia, by coordinating their effector proteins^2^,^3^. Rho-associated kinase (ROCK), which is one of the effector proteins of RhoA, promotes the actin crosslinking activity of myosin II by phosphorylating myosin light chain (MLC)^4^ and myosin phosphatase^5^. Activated myosin promotes actin filament contraction, thereby inducing the cell morphological change, cytokinesis, and apoptosis^6^,^7^. Because the actin-myosin complex, also known as actomyosin, is a key system for cell-fate determination^8^, an understanding of the regulatory mechanisms of actin cytoskeleton dynamics is very important.

PCTAIRE kinase 3/cyclin-dependent kinase 18 (PCTK3/CDK18) is a serine-threonine protein kinase that belongs to the CDK family^9^. Although CDKs are widely expressed in mammalian cells, PCTK3 is expressed in specific tissues and cell types, suggesting that PCTK3 is involved in specific functions in post-mitotic cells^10^. The CDK family requires a specific regulatory subunit and/or post-translational protein modification for full activation^11^. We previously revealed that PCTK3 is activated by two pathways: interaction with cytoplasmic cyclin A and phosphorylation at Ser-12 by protein kinase A (PKA)^12^. Activated PCTK3 phosphorylates retinoblastoma protein (Rb) *in vitro*. Although the activation mechanism of PCTK3 is gradually becoming clear, the downstream signaling pathway of PCTK3 remains unknown. CDK5, a neuronal CDK, controls cell migration via the phosphorylation of actin cytoskeletal proteins, including focal adhesion kinase (FAK), paxillin, and talin^13^,^14^,^15^. PCTK1/CDK16 regulates cytoskeletal rearrangement and neural migration in cooperation with CDK5^16^. A recent study reported that PCTK1 regulates spindle orientation via phosphorylation of Ser-83 on KAP0, a regulatory subunit of PKA^17^. We previously found that knockdown of PCTK3 induced morphological changes and actin accumulation at the leading edges of HEK293T cells. In addition, PCTK3 knockdown increased the phosphorylation of cofilin at Ser-3^12^. The actin depolymerizing activity of cofilin is inactivated by Rho GTPase-mediated phosphorylation at Ser-3^18^,^19^. Thus, our previous findings suggest that PCTK3 may be involved in the regulation of actin cytoskeleton dynamics by controlling the activities of Rho GTPases.

The extracellular matrix (ECM) is a fundamental component of multicellular organisms, and is critically important for many cellular processes, including cell proliferation, differentiation, and motility^20^. Cells are attached to the ECM through cell-surface receptors, such as the integrin family, which stimulates focal adhesion assembly^21^. Focal adhesion is a macromolecular complex consisting of various proteins, including ECM-interacting proteins, integrin-actin crosslinking proteins, tyrosine kinases, and substrates of tyrosine kinases^22^. Focal adhesion is also connected with the actin cytoskeletal network, including actin binding proteins and Rho GTPases^23^. FAK is a cytoplasmic non-receptor tyrosine kinase and plays a crucial role in the regulation of cell motility^24^. ECM-mediated integrin clustering promotes FAK activation and autophosphorylation at Tyr-397, resulting in the Src homology 2 (SH2)-dependent recruitment and binding of the Src family^24^,^25^. Activated FAK also regulates actin polymerization by controlling the balance of Rho GTPases^26^. In contrast, FAK-related non-kinase, which is an autonomously expressed carboxyl-terminal region of FAK, suppresses cell migration and proliferation by interrupting FAK activation^27^. G protein-coupled receptor-induced phosphorylation of FAK at Ser-843 also downregulates FAK activity^28^,^29^. Although it has been demonstrated the importance of FAK in the regulation of cell motility, the regulatory mechanism of FAK is not fully understood.

To obtain additional information on the physiological function of PCTK3, we investigated the signaling pathway of PCTK3 and the actin cytoskeleton. In this study, we determined that PCTK3 regulates cell migration by controlling the balance between RhoA and Rac1 activities. Furthermore, we found that PCTK3 suppresses cell adhesion and spreading by negatively modulating the FAK activity. These findings are valuable for future investigations of PCTK3 function.

## Results

### PCTK3 regulates cell migration by modulating the phosphorylation of cofilin and MLC

**Figure 1:**
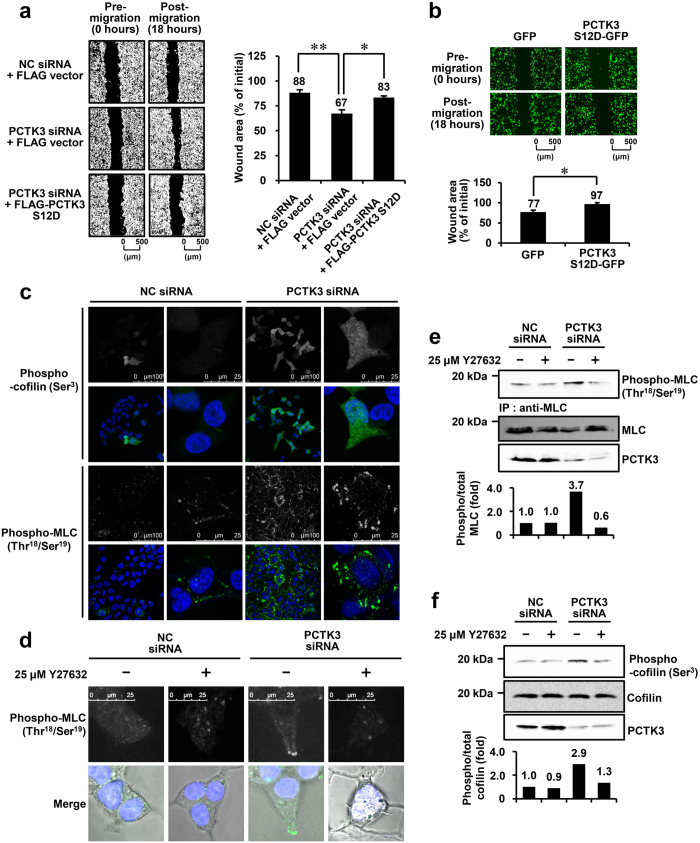
Knockdown of PCTK3 promotes cell migration and the phosphorylation of cofilin and MLC. (**a**) HEK293T cells plated on fibronectin-coated plates were transfected with negative control (NC) or PCTK3 siRNA. Twenty-four hours later, cells were transfected with empty FLAG vector or FLAG-tagged PCTK3 S12D for 22 hours. The cells were serum-starved and treated with 0.5 μg/ml mitomycin C. After 2 hours, confluent monolayer cells were scratched, and images were captured by phase microscopy using a 4× objective lens 0 and 18 hours after the scratch. Quantification of the wound area was performed using Image J. The wound area was calculated as the percentage of the initial wound area. Results are expressed as means ± S.E. from three independent experiments. Statistical significance was determined by one-way ANOVA with Tukey’s multiple comparison test. *p < 0.05 and **p < 0.01. (**b**) HeLa cells were transfected with GFP or PCTK3 S12D-GFP. The cells were serum-starved and treated with mitomycin C. After 2 hours, *in vitro* scratch assay was performed. The images were captured by microscopy using a 4× objective lens. Results are expressed as means ± S.E. from three independent experiments. Statistical significance was determined by Student’s *t*-test. *p < 0.05. (**c**) HEK293T cells were transfected with NC or PCTK3 siRNA for 48 hours. Cells were fixed and incubated with anti-phospho-cofilin (Ser-3) or anti-phospho-MLC (Thr-18/Ser-19) antibodies. Fluorescence for phospho-cofilin and phospho-MLC is shown in *green*. Hoechst nuclear staining is represented in *blue*. All images were obtained using a 63× objective lens. The digitally 4–6 times magnified images of different field-of-views are shown in the right panel. (**d**) PCTK3-knockdown HEK293T cells were serum starved for 6 hours, and were then treated with 25 μM Y27632 for 4 hours. The cells were subjected to immunofluorescence analysis using anti-phospho-MLC antibody. (**e**,**f**) PCTK3-knockdown HEK293T cells were serum starved and were treated with Y27632 for 4 hours. The cell lysates were subjected to immunoprecipitation with anti-MLC antibody. The immunoprecipitates or lysates were analyzed by immunoblotting with anti-phospho-MLC or anti-phospho-cofilin antibodies. Band intensities were quantified using the Image J. The levels of phosphorylated form were normalized to the levels of total proteins.

We previously reported that PCTK3 knockdown induces morphological changes, actin accumulation in lamellipodia, and cofilin inactivation in HEK293T cells^12^. Proteins involved in actin cytoskeletal reorganization, including cofilin and myosin, are thought to be essential for cell membrane extension, migration, and adhesion^1^. Therefore, we examined whether PCTK3 is involved in the regulation of cell migration using an *in vitro* scratch assay. HEK293T cells were plated on fibronectin-coated plate and then transfected with negative control siRNA or PCTK3 siRNA. To exclude the effect of cell proliferation, cells were serum starved and exposed to mitomycin C before scratching. As shown in Fig. 1a, the wound area of post-migration was narrowed to <70% of the pre-migration area in PCTK3-knockdown HEK293T cells, whereas no difference in wound area was observed between pre- and post-migration in control cells. To confirm the specificity of the effect of PCTK3 siRNA, a rescue experiment was performed using an RNAi-resistant mouse PCTK3 S12D, which is a constitutively active mutant of PCTK3^12^. Induction of cell migration in PCTK3-knockdown cells was significantly suppressed by transfection of FLAG-PCTK3 S12D. Furthermore, we examined the effect of forced PCTK3 expression on cell migration in HeLa cells, which only exhibit weak endogenous PCTK3 expression^12^. Overexpression of PCTK3 S12D-GFP significantly suppressed cell migration (Fig. 1b). These results strongly suggest that PCTK3 negatively regulates cell migration.

Cell motility is controlled through reorganization of the actin cytoskeleton. The phosphorylation of cofilin and MLC is an important event in the regulation of actin dynamics^1^. Thus, we examined whether PCTK3 controls the phosphorylation of cofilin and MLC. Immunofluorescence analysis showed that phosphorylated cofilin (Ser-3) and MLC (Thr-18/Ser-19) were increased in PCTK3-knockdown cells when compared with control cells (Fig. 1c and d). Notably, phosphorylated MLC was localized at the leading edge of PCTK3-knockdown cells. The phosphorylation of MLC is also regulated by various actin cytoskeleton-associated proteins, including ROCK. PCTK3 knockdown induced an increase in MLC phosphorylation, and treatment with Y27632, a ROCK-specific inhibitor, reduced MLC phosphorylation to the same level of control cells (Fig. 1d). Similarly, immunoblot analysis confirmed that PCTK3 knockdown-induced MLC (Thr-18/Ser-19) and cofilin (Ser-3) phosphorylation was suppressed by Y27632 treatment (Fig. 1e and f). These results indicate that PCTK3 is involved in the regulation of the RhoA/ROCK signaling pathway.

### PCTK3 negatively regulates the RhoA/ROCK signaling pathway

**Figure 2:**
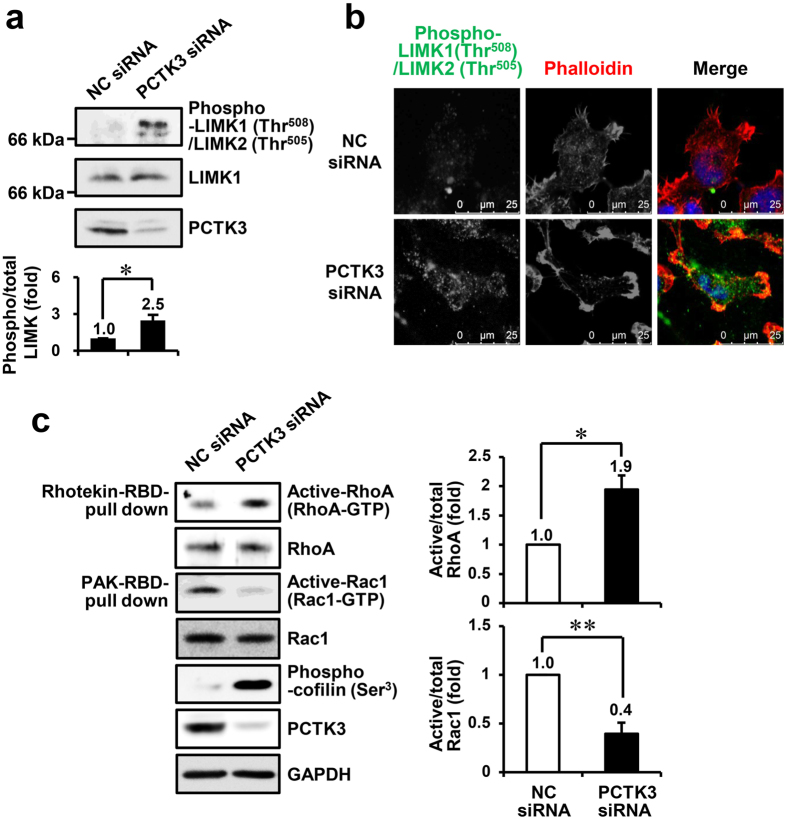
PCTK3 knockdown increases RhoA activity and decreases Rac1 activity. (**a**) Cell lysates of HEK293T cells transfected with negative control (NC) siRNA or PCTK3 siRNA were subjected to immunoblot analysis using anti-phospho-LIMK1 (Thr-508)/LIMK2 (Thr-505), anti-LIMK1, and anti-PCTK3 antibodies. The levels of phosphorylated LIMK1 (Thr-508)/LIMK2 (Thr-505) were normalized to total LIMK levels. The phosphorylation of LIMK1 (Thr-508)/LIMK2 (Thr-505) in NC siRNA-transfected cells were taken as 1. Results are expressed as means ± S.E. from three independent experiments. Statistical significance was determined by Student’s *t*-test. *p < 0.05. (**b**) HEK293T cells plated on poly-L-lysine-coated coverslips were fixed, and then stained with anti-phospho-LIMK1 (Thr-508)/LIMK2 (Thr-505) antibody (*green*) and Alexa 555-conjugated phalloidin (*Red*). Hoechst nuclear staining is represented in *blue*. The images were captured by using a 63× objective lens. (**c**) PCTK3-knockdown cell lysates were incubated with Rhotekin-RBD or PAK-PBD beads. The active RhoA and Rac1 bound to the beads were subjected to immunoblot analysis using anti-RhoA and anti-Rac1 antibodies, respectively. Relative activities of RhoA and Rac1 were normalized by the RhoA and Rac1 protein levels in corresponding cell lysates. The activities of RhoA and Rac1 in NC siRNA-transfected cells were taken as 1. Results are expressed as means ± S.E. from three independent experiments. Statistical significance was determined by Student’s *t*-test. *p < 0.05 and **p < 0.01.

ROCK activates LIM-domain kinase (LIMK) 1 and 2 via phosphorylation at Thr-508 and Thr-505 within the activation loop, respectively, which leads to cofilin phosphorylation^30^. LIMK1 (Thr-508)/LIMK2 (Thr-505) phosphorylation was significantly increased in PCTK3-knockdown cells (Fig. 2a and b), suggesting that PCTK3 controls cell motility via the RhoA/ROCK/LIMK signaling pathway. To confirm the negative effect of PCTK3 on the RhoA/ROCK signaling pathway, we examined RhoA and Rac1 activities in PCTK3-knockdown cells using Rhotekin-RBD and PAK-PBD pull-down methods. As shown in Fig. 2c, PCTK3 knockdown led to RhoA activation and Rac1 inactivation in HEK293T cells, indicating that PCTK3 controls the balance between the RhoA and Rac1 activities.

**Figure 3:**
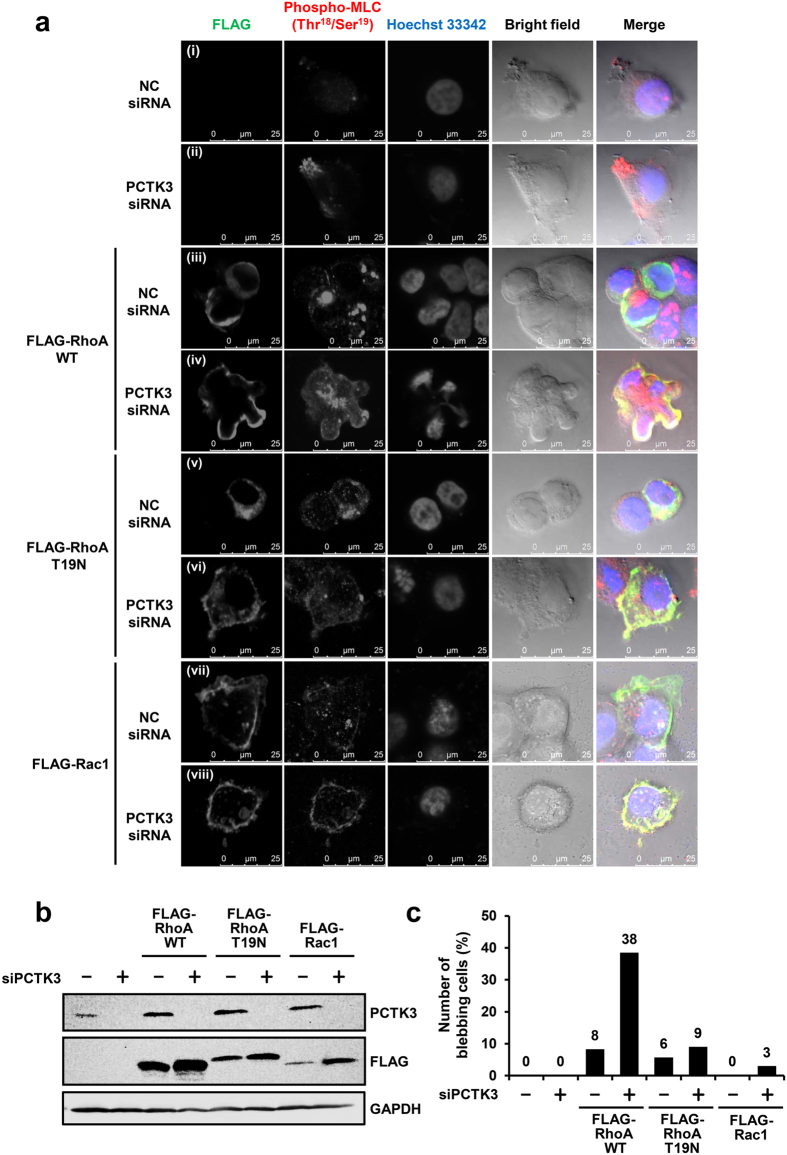
RhoA induces bleb formation in PCTK3-knockdown HEK293T cells. (**a**) HEK293T cells plated on poly-L-lysine-coated coverslips were transfected with NC ((i), (iii), (v), (vii)) or PCTK3 ((ii), (iv), (vi), (viii)) siRNA. After 24 hours, cells were transfected with empty FLAG vector ((i), (ii)), FLAG-tagged RhoA wild type (WT) ((iii), (iv)), T19N mutant ((v), (vi)), or Rac1 ((vii), (viii)) for 12 hours, and then were serum starved for 12 hours. Cells were fixed and stained with anti-FLAG or anti-phospho-MLC (Thr-18/Ser-19) antibodies. Fluorescence for FLAG and phospho-MLC is shown in *green* and *red*, respectively. Hoechst nuclear staining is represented in *blue*. Confocal images were performed using a 63× objective lens. (**b**) The cell lysates were subjected to immunoblot analysis using anti-PCTK3 and anti-FLAG antibodies. (**c**) Total cells and cells with blebs were counted in 6-7 fields randomly selected. At least 30 cells were counted, and the number of cells with blebs was expressed as a percentage of the total number of counted.

The RhoA/ROCK/MLC pathway plays an important role in bleb formation during cell migration, cytokinesis, and apoptosis^4^,^6^,^7^. Rac1 also promotes cytoskeletal reorganization, which leads to the lamellipodia formation^2^,^3^. To verify if RhoA and Rac1 cause morphological changes in PCTK3-knockdown cells, FLAG-tagged wild-type RhoA, RhoA dominant-negative mutant (T19N), or Rac1 were overexpressed in PCTK3-knockdown cells (Fig. 3a and b). Phosphorylated MLC (Thr-18/Ser-19) was concentrated at the leading edge of PCTK3-knockdown cells (Fig. 3a(i) vs. (ii)). In negative control siRNA-transfected HEK293T cells, overexpressed wild-type RhoA was mainly localized in the cytoplasm and slightly promoted MLC phosphorylation, but did not affect cellular and nuclear morphology (Fig. 3a(iii)). Interestingly, we observed membrane bleb formation, colocalization of RhoA and phosphorylated MLC, and abnormal nuclear morphology in RhoA-expressing PCTK3-knockdown cells (Fig. 3a(iv)). Total cells and cells with blebs were counted, and the percentage of cells with blebs was calculated. The number of cells with blebs in RhoA-expressing PCTK3-knockdown cells increased to 38% of the population, whereas those in other cells were lower (<10%) (Fig. 3c). On the other hand, RhoA T19N and Rac1 were localized in the cytoplasm, and did not affect cellular and nuclear morphology or MLC phosphorylation in both control siRNA- and PCTK3 siRNA-transfected cells (Fig. 3a(v–viii)). This finding suggests that MLC phosphorylation and morphological changes are dependent on RhoA activity, and that PCTK3 is involved in the RhoA/ROCK/MLC signaling pathway.

### PCTK3 negatively regulates FAK and Src tyrosine kinase activities

**Figure 4:**
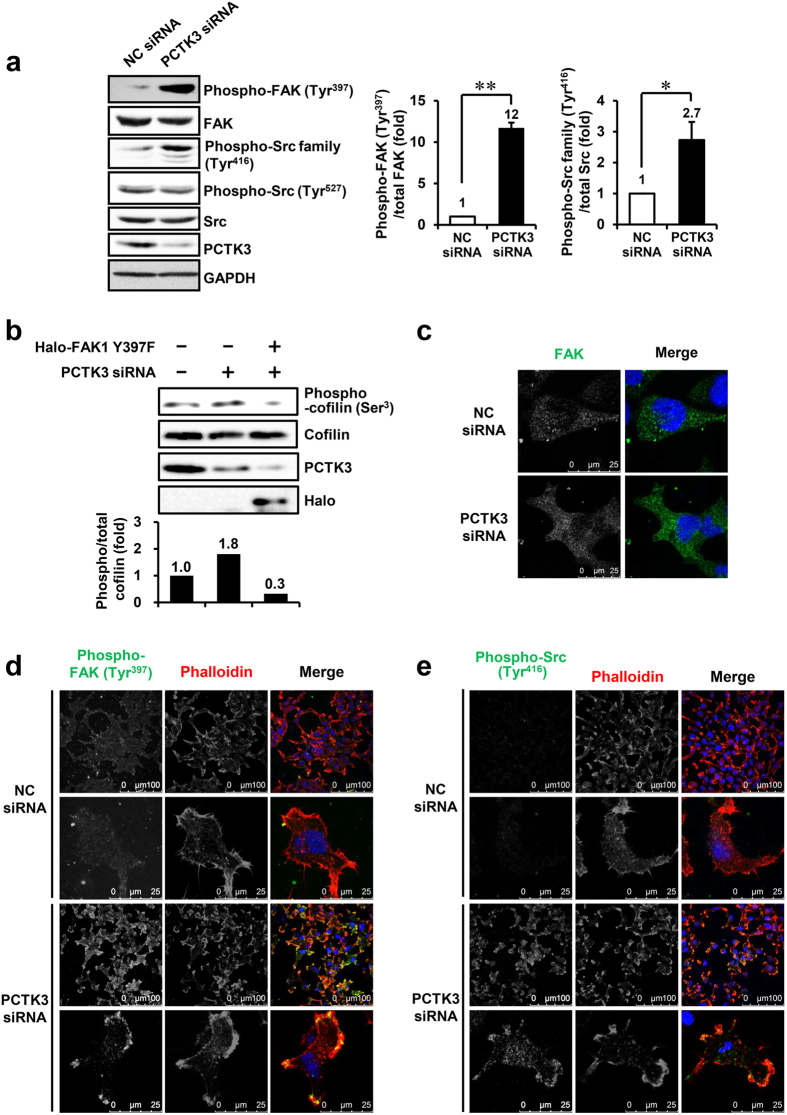
PCTK3 negatively regulates FAK and Src tyrosine kinase activities. (**a**) PCTK3-knockdown cell lysates were subjected to immunoblot analysis using anti-FAK, anti-Src, anti-phospho-FAK (Tyr-397), anti-phospho-Src (Tyr-416), anti-phospho-Src (Tyr-527), and anti-PCTK3 antibodies. Immunoblot intensity of GAPDH was used as a loading control. Relative phosphorylation of FAK and Src were normalized by total FAK and Src levels, respectively. The phosphorylation of FAK and Src in NC siRNA cells was taken as 1. Results are expressed as means ± S.E. from three independent experiments. Statistical significance was determined by Student’s *t*-test. *p < 0.05 and **p < 0.01. (**b**) HEK293T cells plated on poly-L-lysine-coated plates were transfected with negative control (NC) siRNA or PCTK3 siRNA. Twenty-four hours later, cells were transfected with Halo-tagged FAK1 Y397F mutant for 24 hours. The cell lysates were subjected to immunoblot analysis using anti-phospho-cofilin (Ser-3) antibody. Relative phosphorylation of cofilin at Ser-3 was normalized by total cofilin levels. The phosphorylation of cofilin at Ser-3 in NC siRNA-transfected cells were taken as 1. (**c**–**e**) HEK293T cells were plated on poly-L-lysine-coated coverslips. Cells were transfected with NC or PCTK3 siRNA for 48 hours, fixed, and immunostained with anti-FAK (**c**), anti-phospho-FAK (Tyr-397) (**d**), and anti-phospho-Src family (Tyr-416) (**e**) antibodies, and Alexa 555-conjugated phalloidin (*Red*) (**d**,**e**). Hoechst nuclear staining is represented in *blue*. All images were obtained using a 63× objective lens. The digitally 4–6 times magnified images of different field-of-views are shown in the lower panel.

Although RhoA activity is mainly regulated by guanine exchange factors (GEFs) and GTPase activating proteins (GAPs)^3^, it is also regulated by phosphorylation via protein kinases, including PKA^31^. Thus, we examined whether PCTK3 also directly phosphorylates RhoA. An *in vitro* kinase assay revealed that PCTK3 failed to phosphorylate RhoA, although it phosphorylated Rb protein (data not shown), indicating that PCTK3 indirectly regulates RhoA activity. FAK, which is a non-receptor tyrosine kinase and plays a central role in focal adhesion assembly, could increase RhoA to ROCK activation via association with various RhoGEFs^24^,^26^. Therefore, we investigated whether PCTK3 regulates RhoA activity through the FAK and Src signaling pathway. As shown in Fig. 4a, PCTK3 knockdown significantly increased phosphorylation of FAK at Tyr-397 and Src at Tyr-416 but not Tyr-527, which is the CSK (C-terminal Src kinase) phosphorylation site. In addition, a dominant negative mutant of FAK1, FAK1 Y397F, in which the major autophosphorylation site is removed, suppressed cofilin phosphorylation induced by PCTK3 knockdown (Fig. 4b), suggesting that PCTK3 regulates the RhoA/ROCK cascade via regulation of FAK. To confirm that PCTK3 regulates FAK and Src phosphorylation, we performed immunofluorescence staining using anti-phospho-FAK (Tyr-397) and anti-phospho-Src family (Tyr-416) antibodies. FAK was widely localized in the cytoplasm in both control siRNA- and PCTK3 siRNA-transfected cells (Fig. 4c). The phosphorylation of FAK at Tyr-397 was not detected in control cells, whereas puncta of phosphorylated FAK were observed near the leading edge of the cell membrane in PCTK3-knockdown cells (Fig. 4d). Similarly, phosphorylated Src (Tyr-416) accumulated at the leading edge of PCTK3-knockdown cells, although it was not detected in control cells (Fig. 4e). These results suggest that PCTK3 controls the balance of RhoA and Rac1 activities by negatively modulating the FAK and Src signaling pathway.

### PCTK3 suppresses FAK activation during early cell adhesion

**Figure 5:**
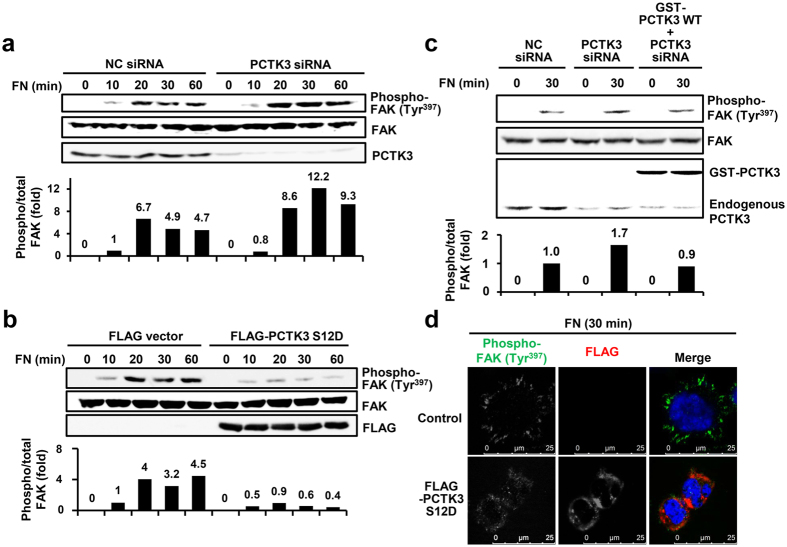
PCTK3 suppresses fibronectin-mediated FAK activation during early cell adhesion. (**a**,**b**) PCTK3 siRNA (**a**) or FLAG-tagged PCTK3 S12D (**b**) transfected HEK293T cells were plated on fibronectin (FN)-coated dishes for indicated times. The cell lysates were subjected to immunoblot analysis with anti-phospho-FAK (Tyr-397), anti-FAK, anti-FLAG, and anti-PCTK3 antibodies. (**c**) HEK293T cells were transfected with NC or PCTK3 siRNA. After 24 hours, cells were transfected with empty GST vector or GST-tagged PCTK3 wild type for 24 hours. Cells were plated on FN-coated dishes for 30 minutes. The cell lysates were subjected to immunoblot analysis with anti-phospho-FAK (Tyr-397), anti-FAK, and anti-PCTK3 antibodies. The levels of phosphorylated FAK (Tyr-397) were normalized to the levels of total FAK. The phosphorylation of FAK (Tyr-397) in fibronectin-stimulated NC siRNA-transfected cells were taken as 1. (**d**) FLAG-PCTK3 S12D-transfected HEK293T cells were plated on FN-coated dishes for 30 minutes. Then, cells were fixed and immunostained with anti-phospho-FAK (Tyr-397) and anti-FLAG antibodies. Fluorescence for phospho-FAK (Tyr-397) and FLAG is shown in *green* and *red*, respectively. Hoechst nuclear staining is represented in *blue*.

FAK autophosphorylation at Tyr-397 is induced by cell adhesion to ECM proteins, such as fibronectin, collagen, and laminin^24^,^32^. We hypothesized that PCTK3 is involved in cell adhesion and spreading because of the negative regulation of FAK autophosphorylation by PCTK3. Transfected HEK293T cells were replated on fibronectin-coated dishes, harvested at the indicated times, and immunoblotted with anti-phospho-FAK (Tyr-397). As shown in Fig. 5a, FAK phosphorylation at Tyr-397 was increased by fibronectin stimulation for 10–60 minutes in negative control siRNA-transfected cells, and FAK autophosphorylation was promoted by PCTK3 knockdown. In contrast, the forced expression of a constitutively active PCTK3 S12D mutant strongly suppressed FAK autophosphorylation (Fig. 5b). Furthermore, PCTK3-knockdown cells were transfected with mouse wild-type PCTK3, and were replated on fibronectin-coated dishes. As shown in Fig. 5c, overexpression of PCTK3 reduced PCTK3 knockdown-induced phosphorylation of FAK at Tyr-397 to the same level of fibronectin-stimulated control cells. Next, we microscopically examined the distribution of phosphorylated FAK in HEK293T cells plated on fibronectin-coated coverslips. Phosphorylated FAK was localized at lamellipodia in control cells, whereas it was decreased and translocated into the cytoplasm in PCTK3 S12D-expressing cells (Fig. 5d). These data indicate that PCTK3 suppresses FAK activation during cell adhesion.

### PCTK3 leads to filopodia formation and a reduction in cell adhesion and spreading in HeLa cells

**Figure 6:**
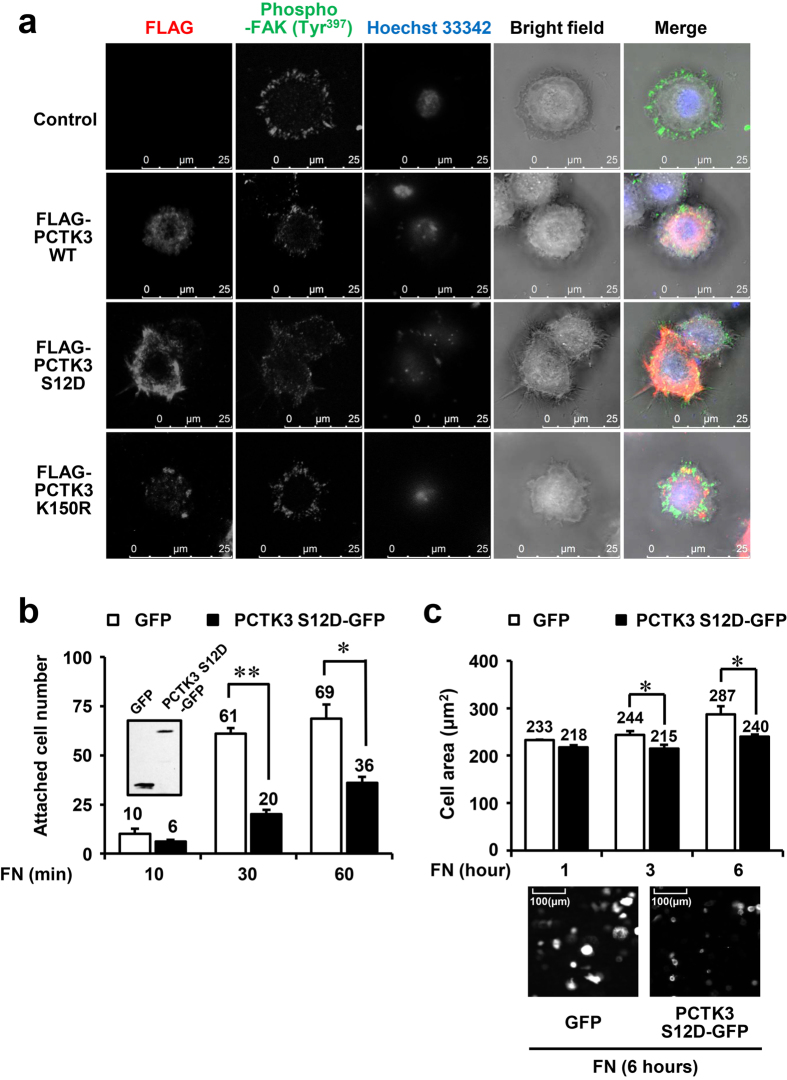
PCTK3 leads to filopodia formation and a reduction in cell adhesion in HeLa cells. (**a**) PCTK3 WT, S12D, or K150R-expressing HeLa cells were plated on fibronectin-coated coverslips for 30 minutes. The cells then were fixed and stained with anti-FLAG and anti-phospho-FAK (Tyr-397) antibodies. Fluorescence for FLAG and phosphor-FAK (Tyr-397) are shown in *red* and *green* respectively. Hoechst nuclear staining is represented in *blue*. The images were captured by using a 63× objective lens. (**b**,**c**) HeLa cells transfected with GFP or PCTK3 S12D-GFP were detached and suspended. Then, cells were plated at a density of 5 × 10^4^ cells per well on fibronectin-coated 12 well plates and incubated for indicated times. Cells were washed with PBS and fixed. The cell area and attached number of GFP expressing cells were counted using a 40× objective lens of IN Cell Analyzer 6000. Results are expressed as means ± S.E. from six independent fields. Statistical significance was determined by Student’s *t*-test. *p < 0.05 and **p < 0.01. Inset: Immunoblot analysis with anti-GFP antibody.

We also analyzed the effect of forced PCTK3 expression on the morphological changes of HeLa cells. After transfection of PCTK3 wild type, S12D, or a kinase dead (K150R) mutant, cells were detached by trypsin, suspended, and plated on fibronectin-coated coverslips for 30 minutes. As shown in Fig. 6a, immunofluorescence staining showed that phosphorylated FAK at Tyr-397 was localized to the lamellipodia of control cells and PCTK3 WT- and K150R-expressing cells. Surprisingly, in PCTK3 S12D-expressing cells, filopodia were observed and phosphorylated FAK (Tyr-397) was translocated to the cytoplasm. We also examined whether PCTK3 activity was associated with cell adhesion. HeLa cells transfected with either GFP or a C-terminal GFP-tagged PCTK3 S12D mutant (PCTK3 S12D-GFP) were detached, suspended, and replated on fibronectin-coated plates for 10–60 minutes. After removing unattached cells by washing, the number of adherent GFP-expressing cells was counted. After 30 and 60 minutes, the number of PCTK3 S12D-GFP-expressing cells was significantly decreased as compared with control GFP-expressing cells (Fig. 6b). Furthermore, we investigated the effect of PCTK3 on cell spreading. HeLa cells transfected with GFP or PCTK3 S12D-GFP were plated on fibronectin-coated plates, and the cell area was measured after 1–6 hours. The forced expression of PCTK3 did not influence the cell area 1 hour after plating. However, the cell area of PCTK3 S12D-expressing cells was slightly but significantly reduced after 3 and 6 hours as compared with that of control cells (Fig. 6c). These results strongly support that PCTK3 has an important function in the regulation of cell morphology, adhesion and spreading.

### PCTK3 forms a complex with FAK

**Figure 7:**
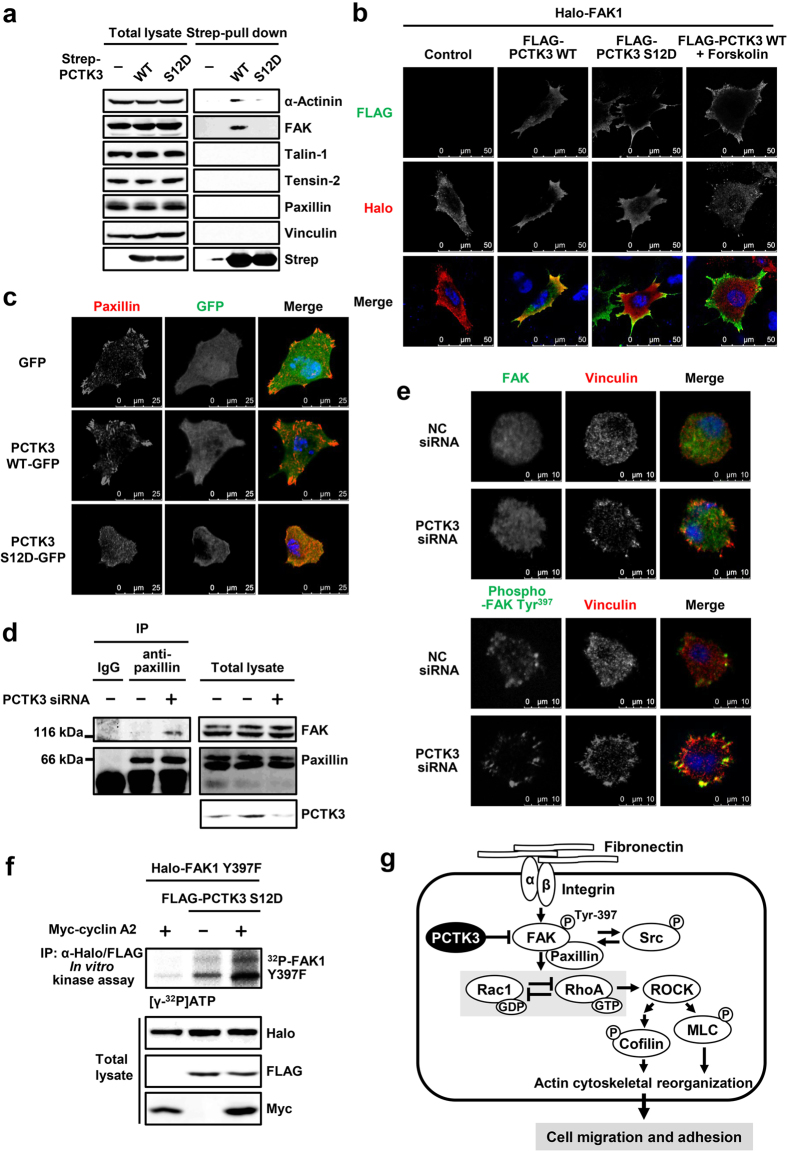
PCTK3 activation induces the dissociation from focal adhesion. (**a**) HEK293T cells were transfected with Strep-tagged PCTK3 wild type (WT) or S12D mutant. Cell lysates were subjected to Strep-pull down assay and immunoblot analysis with anti-α-actinin, anti-FAK, anti-talin-1, anti-tensin-2, anti-paxillin, anti-vinculin, and anti-Strep antibodies. (**b**) HeLa cells were transfected with Halo-tagged FAK1 together with FLAG-tagged PCTK3 WT or S12D. After treatment with 20 μM forskolin or DMSO for 30 minutes, cells were fixed and immunostained with anti-Halo and anti-FLAG antibodies. Fluorescence for Halo and FLAG are shown in *red* and *green*, respectively. Hoechst nuclear staining is represented in *blue*. The images were obtained using a 63× objective lens. (**c**) GFP-, PCTK3 WT-GFP-, or PCTK3 S12D-GFP-expressing HeLa cells were plated on fibronectin-coated coverslips. After 3 hours, cells were fixed and immunostained with anti-paxillin antibody. Fluorescence for GFP and paxillin are shown in *green* and *red*, respectively. Hoechst nuclear staining is represented in *blue*. (**d**) The lysates of PCTK3-knockdown cells were subjected to immunoprecipitation with anti-paxillin antibody or control IgG. The immunoprecipitates or lysates were analyzed by immunoblotting using anti-FAK, anti-paxillin, and anti-PCTK3 antibodies. (**e**) PCTK3 siRNA-transfected HEK293T cells were plated on fibronectin-coated dishes for 30 minutes. Then, cells were fixed and stained with anti-FAK, anti-phospho-FAK (Tyr-397), and anti-vinculin antibodies. Fluorescence for FAK and phospho-FAK (Tyr-397) are shown in *green*. Fluorescence for vinculin is shown in *red*. Hoechst nuclear staining is represented in *blue*. (**f**) FLAG-PCTK3 S12D was expressed with Halo-FAK Y397F in the presence or absence of Myc-cylin A2 in HEK293T cells. After immunoprecipitation using anti-FLAG and anti-Halo antibodies, the precipitated samples were incubated in a kinase buffer containing [γ − ^32^P]ATP for 30 min, and subjected to SDS-PAGE, after which the gel was analyzed by bioimaging analyzer. The expression levels of FLAG-tagged, Halo-tagged, and Myc-tagged proteins were confirmed by immunoblotting with anti-FLAG, anti-Halo, and anti-Myc antibodies, respectively. (**g**) A model of the PCTK3/FAK signaling pathway.

Focal adhesion is formed by aggregation of various proteins, such as integrins, FAK, talin, and vinculin^22^,^33^. Because PCTK3 negatively modulates FAK activity, we predicted that PCTK3 was associated with focal adhesion proteins. To investigate the interaction between PCTK3 and focal adhesion proteins, we performed a pull-down assay. A strep-tagged PCTK3 wild type or S12D mutant was expressed in HEK293T cells, and the cell lysates were incubated with Strep-Tactin beads. PCTK3-bound beads were subjected to immunoblot analysis using antibodies against focal adhesion components. We found that wild-type PCTK3 weakly interacted with FAK and α-actinin, but interactions with other focal adhesion proteins were not detected (Fig. 7a). Interestingly, the constitutively active PCTK3 S12D mutant did not interact with FAK and α-actinin. Immunofluorescence staining also showed that wild-type PCTK3 was colocalized with FAK1 in the cytoplasm, whereas the PCTK3 S12D mutant translocated to the cell membrane and did not colocalize with FAK1 in HeLa cells (Fig. 7b). When wild-type PCTK3-expressing HeLa cells were treated with the adenylyl cyclase activator, forskolin, wild-type PCTK3 translocated to the cell membrane, resulting in the reduction in PCTK3 and FAK1 colocalization. These results suggest that activated PCTK3 is dissociated from FAK.

Furthermore, we examined whether PCTK3 affects the formation of focal adhesions. HeLa cells transfected with GFP, wild-type PCTK3-GFP, or PCTK3 S12D-GFP, were plated on fibronectin-coated plates. After 3 hours, cells were immunostained with anti-paxillin antibody. As shown in Fig. 7c, PCTK3 S12D-expressing cells exhibited slower cell spreading as compared with control and wild-type PCTK3-expressing cells. In addition, the number of paxillin clusters was decreased in PCTK3 S12D-expressing cells (19 ± 2.0 clusters per cell) as compared with control GFP- (35 ± 1.1 clusters) and wild-type PCTK3-expressing cells (30 ± 1.7 clusters). This result strongly suggests that activation of PCTK3 is involved in the formation of focal adhesion complexes. We also investigated the interaction of FAK with adhesion proteins such as paxillin, in PCTK3-knockdown cells. Cell lysates from PCTK3-knockdown cells were immunoprecipitated with anti-paxillin antibody, and the immunoprecipitates were immunoblotted with anti-FAK antibody. As shown in Fig. 7d, PCTK3 knockdown promoted the interaction between FAK and paxillin in HEK293T cells. Immunostaining showed that phosphorylated FAK at Tyr-397 was localized in the peripheral area of both control and PCTK3-knockdown cells, whereas total FAK (phosphorylated and unphosphorylated) diffused throughout the cell (Fig. 7e). PCTK3 knockdown induced the translocation of vinculin from the cytoplasm to the peripheral area, and membrane-localized vinculin was colocalized with phosphorylated FAK. These data indicate the involvement of PCTK3 in the regulation of focal adhesion formation.

Finally, we investigated whether PCTK3 directly phosphorylates FAK. As shown in Fig. 7f, an *in vitro* kinase assay revealed that a FAK1 mutant lacking the autophosphorylation site, FAK1 Y397F, was phosphorylated by constitutively active PCTK3 S12D, and was more strongly phosphorylated in the presence of the cyclin A2, suggesting that PCTK3 may directly phosphorylate FAK. These results strongly support that PCTK3 is complexed with FAK, and that its interaction is suppressed by PCTK3 activation.

## Discussion

Although PCTK3 has been suggested to play a role in post-mitotic cells^10^, the physiological function and downstream signaling pathway of PCTK3 remains unclear. We previously reported that PCTK3 knockdown in HEK293T cells promoted the accumulation of polymerized actin in peripheral areas and cofilin phosphorylation^12^. Furthermore, overexpression of a constitutively active PCTK3 mutant effectively suppressed cofilin phosphorylation, suggesting that PCTK3 was involved in actin cytoskeleton organization. In this study, we revealed that PCTK3 knockdown increased cell motility in HEK293T cells. In addition, overexpression of the constitutively active PCTK3 significantly suppressed cell migration of HeLa cells, strongly suggesting that PCTK3 negatively regulates cell migration. Cofilin is inactivated by phosphorylation at Ser-3 by LIMK. In many cases, phosphorylation of the actin-depolymerizing protein cofilin results in a reduction in cell motility. However, a previous report showed that VEGF-A induced cell migration via LIMK1 activation and cofilin phosphorylation^34^. Because it is likely that the precise and balanced regulation of cofilin phosphorylation is important for cell migration, PCTK3 knockdown may disrupt it and trigger cell migration. On the other hand, we also demonstrated that the phosphorylation of MLC at Thr-18 and Ser-19 was increased in PCTK3-knockdown cells, and MLC phosphorylation was suppressed by the ROCK inhibitor Y27632. MLC phosphorylation promotes the contractility of actomyosin in cells, leading to enhanced cell motility. PCTK3 may regulate cell motility via phosphorylation of MLC. In many cell types, MLC is phosphorylated by RhoA-activated ROCK, which results in actin cytoskeletal rearrangement^4^. Therefore, we predicted that PCTK3 regulated the actin cytoskeleton via the RhoA/ROCK/MLC signaling pathway. The activity of Rho family GTPases, including RhoA are strictly controlled by GEFs^35^, GAPs^36^, and guanine nucleotide dissociation inhibitors (GDIs)^37^. The GTP-bound active form of RhoA was greatly increased in PCTK3-knockdown cells, whereas Rac1, another member of the Rho small GTPase family, was suppressed. RhoA and Rac1 often antagonistically regulate each other during cell migration^38^,^39^,^40^. For example, RhoA/ROCK induces bleb formation by activating filamin A-binding RhoGAP, which then decreases Rac1 activity^38^. Alternatively, RhoA is downregulated by the Rac1-mediated activation of p190 RhoGAP and inactivation of GEF-H1^39^,^40^. The balance of Rho GTPases plays a pivotal role in the coordination of various biological processes, including cell motility. To form membrane protrusions that coordinate cell motility, the local activation of Rac and the inactivation of Rho at the leading edge are necessary. The imbalance of RhoA and Rac1 activities causes abnormal membrane protrusion. We found that RhoA overexpression significantly increased MLC phosphorylation and triggered bleb formation in PCTK3-knockdown HEK293T cells, whereas RhoA-transfected control cells showed no morphological changes. We suggest that PCTK3 knockdown may accelerate the imbalance of RhoA and Rac1 activities, resulting in bleb formation. The formation of lamellipodia membrane ruffling and membrane localization of phosphorylated FAK in response to adhesion to fibronectin were observed in HeLa cells. Contrary to this observation, increased filopodia density and cytosolic translocation of phosphorylated FAK were observed in PCTK3 S12D-expressing cells. We speculate that excessive formation of filopodia by constitutively active PCTK3 is due to the disruption of the balance between RhoA, Rac1, and cdc42 activities. Furthermore, we showed that PCTK3 reduced the ability of cell adhesion and spreading in HeLa cells. Cell adhesion is important for the organization and maintenance of specific tissues and organs, and is regulated by various molecules located on the cell surface, such as cadherin and integrin^21^. Because the invasiveness of carcinoma cells is controlled by E-cadherin-mediated cell-cell adhesion and integrin-mediated ECM-cell adhesion^41^,^42^, it is expected that PCTK3 may regulate cancer motility.

ECM-integrin interactions promote FAK phosphorylation at Tyr-397, which is required for the assembly of focal adhesions^24^. We demonstrated that PCTK3 suppressed attachment-induced phosphorylation of FAK at Tyr-397, which reduces cell migration and adhesion via the RhoA/ROCK inactivation (Fig. 7g). Therefore, we predict that PCTK3 suppresses the interaction between FAK and focal adhesion proteins, including integrins, paxillin and vinculin. As expected, overexpression of constitutively active PCTK3 decreased the number of paxillin clusters as compared with control and wild type PCTK3, strongly supporting that activation of PCTK3 is involved in the formation of focal adhesion complexes. FAK phosphorylation at Tyr-397 is also regulated by receptor tyrosine kinases, such as EGF receptor, PDGF receptor, and EphB receptor^24^,^43^,^44^. EphB receptor induces dendritic filopodia via FAK and RhoA activation^43^. On the other hand, FAK activity is also regulated by intracellular signaling pathways, such as serine phosphorylation, in an integrin-independent manner. CDK5 phosphorylates FAK at Ser-732, which promotes FAK autophosphorylation and neural migration^13^. Ser-732 phosphorylation of FAK is also induced by ROCK activation in response to VEGF, and is important for focal adhesion assembly and endothelial cell migration^45^. We determined that the constitutively active PCTK3 S12D mutant phosphorylated FAK1 *in vitro*. FAK1 has eight putative CDK phosphorylation sites (Ser/Thr-Pro) (Thr-13, Ser-29, Ser-274, Ser-443, Ser-722, Ser-732, Ser-893, and Ser-910), suggesting that FAK1 is one of the most likely substrates for PCTK3. In addition, focal adhesion proteins, such as paxillin and vinculin, are also regulated by phosphorylation^13^,^14^,^15^,^46^. For example, CDK5 directly phosphorylates paxillin at Ser-244 and reduces the interaction between FAK and paxillin^14^. Paxillin is also phosphorylated by cytoplasmic cyclin D1/CDK4 at Ser-83 and Ser-178, resulting in the activation of Rac1 and promoting cell invasion^47^. Because active PCTK3 was released from FAK and α-actinin, focal adhesion proteins may be a putative substrate of PCTK3. Further analyses are needed to resolve the true signaling pathway between PCTK3 and FAK.

The catalytic activity of PCTK3 is enhanced by PKA phosphorylation at Ser-12^12^. PKA modulates the actin cytoskeleton by directly phosphorylating actin-associated proteins, such as RhoA, PAK, and VASP^31^,^48^. In addition, PKA mediated-protein tyrosine phosphatase 1B activation promotes the disruption of focal adhesion and dephosphorylation of FAK Tyr-397 in adult rat cardiac fibroblasts^49^. These findings suggest that PKA negatively regulates FAK activity via activation of PCTK3 and/or protein tyrosine phosphatase 1B. Furthermore, PCTK3 activity is activated by association with cyclin A^12^. We demonstrated that FAK1 was strongly phosphorylated by the cyclin A/PCTK3 S12D complex. It has been well established that cyclin A plays a central role in cell cycle progression in somatic cells by activating CDK1 and CDK2^50^. Previous studies showed that cyclin A controls cell invasion by directly activating RhoA^51^,^52^, although little is known about the role of cyclin A in the regulation of cell motility. These findings suggest that cyclin A has dual functions in the regulation of cell motility via direct RhoA activation and indirect PCTK3-mediated FAK inactivation.

In summary, we identified a novel regulatory mechanism of cell motility via PCTK3. Cell migration is a typical cell function associated with various physiological phenomena, such as organogenesis, wound healing, cancer invasion and metastasis. To overcome the risk for carcinogenesis and malignant progression, a better understanding of the regulatory mechanisms of cell motility is needed to develop new anticancer agents. Further investigation, including animal research, is needed to verify that PCTK3 has the potential to be a cancer therapeutic target.

## Materials and Methods

### Antibodies

Anti-phospho-cofilin (Ser-3), anti-cofilin, anti-phospho-MLC (Thr-18/Ser-19), anti-MLC, anti-phopsho-LIMK1 (Thr-508)/LIMK2 (Thr-505), anti-LIMK1, anti-phospho-Src family (Tyr-416), anti-phospho-Src (Tyr-527), anti-Src, anti-phospho-FAK (Tyr-397), anti-FAK, anti-α-actinin, anti-paxillin, anti-talin-1, and anti-tensin-2 antibodies were purchased from Cell Signaling Technology. Anti-PCTK3 and anti-vinculin antibodies were from Santa Cruz Biotechnology. Anti-FLAG (M2) antibody was form Sigma-Aldrich. Anti-Strep antibody was from Qiagen. Anti-Halo antibody was from Promega. Anti-GAPDH antibody was from Wako Pure Chemical Industries. Anti-RhoA and Rac1 antibodies and Alexa-555 conjugated-phalloidin were from Cytoskeleton. Anti-Myc antibody was from Enzo Life Sciences.

### Cell culture, DNA transfection and RNA interference

HEK293T cells and HeLa cells were maintained in Dulbecco’s modified Eagle’s medium (DMEM) supplemented with 10% fetal bovine serum (FBS), 100 units/ml penicillin, and 100 μg/ml streptomycin at 37 °C in 5% CO_2_. Cells were transfected with various expression vectors or siRNA using Lipofectamine 2000 (Invitrogen/Thermo Fisher Scientific) according to the manufacturer’s instructions. For RNA interference, synthetic siRNA oligonucleotide specific for PCTK3 (PCTK3 siRNA [ID# SASI_Hs02_00334101]) was obtained from Sigma-Aldrich. A MISSION siRNA Universal Negative Control was used as the negative control. For double transfection, the second transfection with various vectors was performed after transfection with siRNAs for 24 hours.

### Immunofluorescence analysis

Immunofluorescence analysis was performed as previously described^12^. In brief, HEK293T cells grown on poly-L-lysine-coated chamber slides were transfected with various expression plasmids or siRNA, and incubated for 24 or 48 hours. In another case, FLAG-PCTK3 expressing HEK293T or HeLa cells were suspended in DMEM with 10% FBS for 30 minutes at 37 °C. Then cells were plated on the fibronectin (Sigma-Aldrich)-coated dishes and incubated for 30 min. Cells were fixed for 20 min in 3.7% formaldehyde, permeabilized for 5 min in 0.1% Triton X-100, and then blocked in 5% BSA for 30 min at room temperature. Cells were incubated with various antibodies in 5% BSA overnight at 4 °C, followed by incubation for 1 h with goat anti-IgG directly conjugated to Alexa Fluor 488 or Alexa Fluor 555 (Molecular Probes/Thermo Fisher Scientific). The slides were washed thoroughly with phosphate-buffered saline (PBS) and mounted in fluorescent mounting medium (Vectashield; Vector Laboratories). Fluorescent images were obtained using a confocal laser-scanning microscope (Leica TCS-SP5) or an IN Cell Analyzer 6000 system (GE Healthcare). Cell number and cell area of GFP-expressing cells were analyzed using IN Cell Investigator 1.6 (GE Healthcare).

### Pull-down and co-immunoprecipitation assays

Pull-down and co-immunoprecipitation assays were performed as previously described^12^. Cells were lysed in an ice-cold TNE buffer (20 mM Tris-HCl, pH 7.5, 150 mM NaCl, 0.5% Nonidet P-40, and 1 mM EDTA) supplemented with protease inhibitors (10 μg/ml leupeptin and 10 μg/ml aprotinin). The cell extracts were clarified by centrifugation at 10,000 × *g* at 4 °C for 10 min. For pull-down assay, equal protein amounts of the lysates were incubated with Strep-Tactin Sepharose (IBA GmbH) at 4 °C overnight. For immunoprecipitation, lysates were incubated with anti-paxillin antibody in the presence of protein G Sepharose (GE Healthcare) overnight at 4 °C. The beads were washed 4 times with wash buffer (20 mM Tris-HCl, pH 7.5, 150 mM NaCl, 0.1% Nonidet P-40, and 1 mM EDTA). Precipitated proteins were subjected to immunoblot analysis. The images were captured using an LAS-4000 image analyzer (Fuji Film). Uncropped scan for the main figures is presented in Supplementary Fig. 1-6

The RhoA and Rac1 activities were measured by RhoA Pull-down Activation Assay Biochem Kit and Rac1 Pull-down Activation Assay Biochem Kit (Cytoskeleton), respectively, according to the manufacturer’s instructions. Cells were lysed in ice-cold cell lysis buffer (50 mM Tris-HCl, pH7.5, 10 mM MgCl_2_, 40 mM NaCl, 62 μg/ml leupeptin, 62 μg/ml pepstatin A, 14 mg/ml benzamidine, and 12 mg/ml tosyl arginine methyl ester). Cell lysates were centrifuged at 10,000 × *g*, 4 °C for 1 min. Equal protein amounts of the lysates (300–800 μg) were incubated with Rhotekin-RBD or PAK-PBD protein agarose beads at 4 °C on a rotator for 2 hours. After incubation, the beads were washed with wash buffer (25 mM Tris-HCl, pH 7.5, 10 mM MgCl_2_, 0.5 M NaCl, and 2% Igepal). The amount of bound RhoA or Rac1 was analyzed by immunoblotting using anti-RhoA and anti-Rac1 antibodies, and normalized to the total RhoA or Rac1 content of cell lysates.

### *In vitro* kinase assay

*In vitro* kinase assay was carried out as previously described^12^. HEK293T cells transfected with pFLAG-PCTK3 S12D and pHalo-FAK1 Y397F were harvested in lysis buffer. Cell lysates were incubated with anti-FLAG and anti-Halo antibodies together with protein G Sepharose (GE Healthcare) overnight at 4 °C. After washing, the kinase reaction was carried out by resuspending the complexes in 100 μl of kinase buffer (50 mM Tris–HCl pH 7.5, 20 mM magnesium acetate, 50 μM ATP, 2 μCi [γ − ^32^P]ATP, and phosphatase inhibitor mixture (Nacalai Tesque)) and incubating for 30 min at 30 °C. Immunocomplexes were released by heating at 95 °C in SDS sample buffer and subjected to SDS-PAGE, and phosphorylated proteins were visualized by FLA-9000 Fluorescent Image Analyzer (Fuji Film).

### *In vitro* scratch assay

HEK293T cells were plated at 1–5 × 10^4^ cells/well on a fibronectin-coated 12-well plate, and were transfected with negative control siRNA or PCTK3 siRNA. Twenty-four hours later, cells were transfected with empty FLAG vector or FLAG-tagged mouse PCTK3 S12D mutant for 24 hours. Cells were serum-starved and treated with 0.5 μg/ml mitomycin C. After 2 hours, the confluent monolayers were scratched with a yellow pipette tip, and further incubated for 18 hours. Images of cells migrated into the scratch were taken on an inverted microscope, and were quantified using ImageJ software (NIH).

### Statistical analysis

All experiments were performed multiple times to confirm their reproducibility. Data were expressed as the mean ± standard error (S.E.), and statistical analysis was performed by Student’s *t*-test or one-way analysis of variance (ANOVA) with Tukey’s multiple comparison test using GraphPad Prism (GraphPad Software). *P* values < 0.05 were considered statistically significant.

## Additional Information

**How to cite this article:** Matsuda, S. *et al*. PCTK3/CDK18 regulates cell migration and adhesion by negatively modulating FAK activity. *Sci. Rep.* **7**, 45545; doi: 10.1038/srep45545 (2017).

**Publisher's note:** Springer Nature remains neutral with regard to jurisdictional claims in published maps and institutional affiliations.

## Supplementary Material

Supplementary Figures

## References

[b1] Pollard T. D. & Cooper J. A. Actin, a central player in cell shape and movement. Science 326, 1208–1212 (2009). 19965462 10.1126/science.1175862 PMC3677050

[b2] Hall A. Rho GTPases and the actin cytoskeleton. Science 279, 509–514 (1998). 9438836 10.1126/science.279.5350.509

[b3] Hall A. Rho family GTPases. Biochem. Soc. Trans. 40, 1378–1382 (2012). 23176484 10.1042/BST20120103

[b4] Amano M. . Phosphorylation and activation of myosin by Rho-associated kinase (Rho-kinase). J. Biol. Chem. 271, 20246–20249 (1996). 8702756 10.1074/jbc.271.34.20246

[b5] Kimura K., Ito M., Amano M. & Chihara K. Regulation of myosin phosphatase by Rho and Rho-associated kinase (Rho-kinase). Science 273, 245 (1996). 8662509 10.1126/science.273.5272.245

[b6] Matsumura F. Regulation of myosin II during cytokinesis in higher eukaryotes. Trends Cell Biol. 15, 371–377 (2005). 15935670 10.1016/j.tcb.2005.05.004

[b7] Mills J. C., Stone N. L., Erhardt J. & Pittman R. N. Apoptotic membrane blebbing is regulated by myosin light chain phosphorylation. J. Cell Biol. 140, 627–636 (1998). 9456322 10.1083/jcb.140.3.627 PMC2140178

[b8] Murrell M., Oakes P. W., Lenz M. & Gardel M. L. Forcing cells into shape: the mechanics of actomyosin contractility. Nat. Rev. Mol. Cell Biol. 16, 486–498 (2015). 26130009 10.1038/nrm4012 PMC7443980

[b9] Malumbres M. . Cyclin-dependent kinases: a family portrait. Nat. Cell Biol. 11, 1275–1276 (2009). 19884882 10.1038/ncb1109-1275 PMC2914104

[b10] Cole A. R. PCTK proteins: the forgotten brain kinases? Neurosignals 17, 288–297 (2009). 19816065 10.1159/000231895

[b11] Malumbres M. & Barbacid M. Mammalian cyclin-dependent kinases. Trends Biochem. Sci. 30, 630–641 (2005). 16236519 10.1016/j.tibs.2005.09.005

[b12] Matsuda S. . PCTAIRE kinase 3/cyclin-dependent kinase 18 is activated through association with cyclin A and/or phosphorylation by protein kinase A. J. Biol. Chem. 289, 18387–18400 (2014). 24831015 10.1074/jbc.M113.542936 PMC4140294

[b13] Xie Z., Sanada K., Samuels B. A., Shih H. & Tsai L. H. Serine 732 phosphorylation of FAK by Cdk5 is important for microtubule organization, nuclear movement, and neuronal migration. Cell 114, 469–482 (2003). 12941275 10.1016/s0092-8674(03)00605-6

[b14] Miyamoto Y. . Cdk5 regulates differentiation of oligodendrocyte precursor cells through the direct phosphorylation of paxillin. J. Cell Sci. 120, 4355–4366 (2007). 18042622 10.1242/jcs.018218

[b15] Huang C. . Talin phosphorylation by Cdk5 regulates Smurf1-mediated talin head ubiquitylation and cell migration. Nat. Cell Biol. 11, 624–630 (2009). 19363486 10.1038/ncb1868 PMC2714540

[b16] Mokalled M. H., Johnson A., Kim Y., Oh J. & Olson E. N. Myocardin-related transcription factors regulate the Cdk5/Pctaire1 kinase cascade to control neurite outgrowth, neuronal migration and brain development. Development 137, 2365–2374 (2010). 20534669 10.1242/dev.047605 PMC2889604

[b17] Iwano S. . PCTK1 Regulates Integrin-Dependent Spindle Orientation via Protein Kinase A Regulatory Subunit KAP0 and Myosin X. Mol. Cell. Biol. 35, 1197–1208 (2015). 25605337 10.1128/MCB.01017-14 PMC4355539

[b18] Moriyama K., Iida K. & Yahara I. Phosphorylation of Ser-3 of cofilin regulates its essential function on actin. Genes Cells 1, 73–86 (1996). 9078368 10.1046/j.1365-2443.1996.05005.x

[b19] Ridley A. J. Rho GTPases and actin dynamics in membrane protrusions and vesicle trafficking. Trends Cell Biol. 16, 522–529 (2006). 16949823 10.1016/j.tcb.2006.08.006

[b20] Theocharis A. D., Skandalis S. S., Gialeli C. & Karamanos N. K. Extracellular matrix structure. Adv. Drug Deliv. Rev. 97, 4–27 (2016). 26562801 10.1016/j.addr.2015.11.001

[b21] Gumbiner B. M. Cell adhesion: the molecular basis of tissue architecture and morphogenesis. Cell 84, 345–357 (1996). 8608588 10.1016/s0092-8674(00)81279-9

[b22] Zaidel-Bar R., Cohen M., Addadi L. & Geiger B. Hierarchical assembly of cell–matrix adhesion complexes. Biochem. Soc. Trans. 32, 416–420 (2004). 15157150 10.1042/BST0320416

[b23] Nobes C. D. & Hall A. Rho, rac, and cdc42 GTPases regulate the assembly of multimolecular focal complexes associated with actin stress fibers, lamellipodia, and filopodia. Cell 81, 53–62 (1995). 7536630 10.1016/0092-8674(95)90370-4

[b24] Mitra S. K., Hanson D. A. & Schlaepfer D. D. Focal adhesion kinase: in command and control of cell motility. Nat. Rev. Mol. Cell Biol. 6, 56–68 (2005). 15688067 10.1038/nrm1549

[b25] Schaller M. D. . Autophosphorylation of the focal adhesion kinase, pp125FAK, directs SH2-dependent binding of pp60src. Mol. Cell. Biol. 14, 1680–1688 (1994). 7509446 10.1128/mcb.14.3.1680-1688.1994 PMC358526

[b26] Tomar A. & Schlaepfer D. D. Focal adhesion kinase: switching between GAPs and GEFs in the regulation of cell motility. Curr. Opin. Cell. Biol. 21, 676–683 (2009). 19525103 10.1016/j.ceb.2009.05.006 PMC2754589

[b27] Richardson A. & Parsons J. T. A mechanism for regulation of the adhesion-associated protein tyrosine kinase pp125FAK. Nature 380, 538–540 (1996). 8606775 10.1038/380538a0

[b28] Jiang X., Sinnett-Smith J. & Rozengurt E. Differential FAK phosphorylation at Ser-910, Ser-843 and Tyr-397 induced by angiotensin II, LPA and EGF in intestinal epithelial cells. Cell. Signal. 19, 1000–1010 (2007). 17240116 10.1016/j.cellsig.2006.11.004 PMC1868572

[b29] Jacamo R., Jiang X., Lunn J. A. & Rozengurt E. FAK phosphorylation at Ser-843 inhibits Tyr-397 phosphorylation, cell spreading and migration. J. Cell. Physio. 210, 436–444 (2007). 10.1002/jcp.20870 17096371

[b30] Ohashi K. . Rho-associated kinase ROCK activates LIM-kinase 1 by phosphorylation at threonine 508 within the activation loop. J. Biol. Chem. 275, 3577–3582 (2000). 10652353 10.1074/jbc.275.5.3577

[b31] Lang P. . Protein kinase A phosphorylation of RhoA mediates the morphological and functional effects of cyclic AMP in cytotoxic lymphocytes. EMBO J. 15, 510–519 (1996). 8599934 PMC449969

[b32] Boudreau N. J. & Jones P. L. Extracellular matrix and integrin signalling: the shape of things to come. Biochem. J. 339, 481–488 (1999). 10215583 PMC1220180

[b33] Wozniak M. A., Modzelewska K., Kwong L. & Keely P. J. Focal adhesion regulation of cell behavior. *Biochim. Biophys. Acta Mol*. Cell Res. 1692, 103–119 (2004). 10.1016/j.bbamcr.2004.04.007 15246682

[b34] Kobayashi M. . MAPKAPK-2-mediated LIM-kinase activation is critical for VEGF-induced actin remodeling and cell migration. EMBO J. 25, 713–726 (2006). 16456544 10.1038/sj.emboj.7600973 PMC1383554

[b35] Rossman K. L., Der C. J. & Sondek J. GEF means go: turning on RHO GTPases with guanine nucleotide-exchange factors. Nat. Rev. Mol. Cell Biol. 6, 167–180 (2005). 15688002 10.1038/nrm1587

[b36] Moon S. Y. & Zheng Y. Rho GTPase-activating proteins in cell regulation. Trends Cell Biol. 13, 13–22 (2003). 12480336 10.1016/s0962-8924(02)00004-1

[b37] DerMardirossian C. & Bokoch G. M. GDIs: central regulatory molecules in Rho GTPase activation. Trends Cell Biol. 15, 356–363 (2005). 15921909 10.1016/j.tcb.2005.05.001

[b38] Ohta Y., Hartwig J. H. & Stossel T. P. FilGAP, a Rho-and ROCK-regulated GAP for Rac binds filamin A to control actin remodelling. Nat. Cell Biol. 8, 803–814 (2006). 16862148 10.1038/ncb1437

[b39] Wildenberg G. A. . B. p120-catenin and p190RhoGAP regulate cell-cell adhesion by coordinating antagonism between Rac and Rho. Cell 127, 1027–1039 (2006). 17129786 10.1016/j.cell.2006.09.046

[b40] Zenke F. T. . p21-activated kinase 1 phosphorylates and regulates 14-3-3 binding to GEF-H1, a microtubule-localized Rho exchange factor. J. Biol. Chem. 279, 18392–18400 (2004). 14970201 10.1074/jbc.M400084200

[b41] Frixen U. H. . E-cadherin-mediated cell-cell adhesion prevents invasiveness of human carcinoma cells. J. Cell Biol. 113, 173–185 (1991). 2007622 10.1083/jcb.113.1.173 PMC2288921

[b42] Hood J. D. & Cheresh D. A. Role of integrins in cell invasion and migration. Nat. Rev. Cancer 2, 91–100 (2002). 12635172 10.1038/nrc727

[b43] Sieg D. J. . FAK integrates growth-factor and integrin signals to promote cell migration. Nat. Cell Biol. 2, 249–256 (2000). 10806474 10.1038/35010517

[b44] Moeller M. L., Shi Y., Reichardt L. F. & Ethell I. M. EphB receptors regulate dendritic spine morphogenesis through the recruitment/phosphorylation of focal adhesion kinase and RhoA activation. J. Biol. Chem. 281, 1587–1598 (2006). 16298995 10.1074/jbc.M511756200

[b45] Le Boeuf F., Houle F., Sussman M. & Huot J. Phosphorylation of Focal Adhesion Kinase (FAK) on Ser732 Is Induced by Rho-dependent Kinase and Is Essential for Proline-rich Tyrosine Kinase-2–mediated Phosphorylation of FAK on Tyr407 in Response to Vascular Endothelial Growth Factor. Mol. Biol. Cell 17, 3508–3520 (2006). 16760434 10.1091/mbc.E05-12-1158 PMC1525237

[b46] Perez-Moreno M., Avila A., Islas S., Sanchez S. & Gonzalez-Mariscal L. Vinculin but not alpha-actinin is a target of PKC phosphorylation during junctional assembly induced by calcium. J. Cell Sci. 111, 3563–3571 (1998). 9811570 10.1242/jcs.111.23.3563

[b47] Fusté N. P. . Cytoplasmic cyclin D1 regulates cell invasion and metastasis through the phosphorylation of paxillin. Nat. Commun. 7, 11581; doi: 10.1038/ncomms11581 (2016). 27181366 PMC4873647

[b48] Howe A. K. Regulation of actin-based cell migration by cAMP/PKA. Biochim. Biophys. Acta -Mol. Cell. Res. 1692, 159–174 (2004). 10.1016/j.bbamcr.2004.03.005 15246685

[b49] Swaney J. S. . Focal adhesions in (myo) fibroblasts scaffold adenylyl cyclase with phosphorylated caveolin. J. Biol. Chem. 281, 17173–17179 (2006). 16618703 10.1074/jbc.M513097200

[b50] Yam C. H., Fung T. K. & Poon R. Y. C. Cyclin A in cell cycle control and cancer. Cell. Mol. Life Sci. 59, 1317–1326 (2002). 12363035 10.1007/s00018-002-8510-y PMC11337442

[b51] Wegiel B. . Multiple cellular mechanisms related to cyclin A1 in prostate cancer invasion and metastasis. J. Natl. Cancer Inst. 100, 1022–1036 (2008). 18612129 10.1093/jnci/djn214 PMC2467435

[b52] Arsic N. . A novel function for Cyclin A2: control of cell invasion via RhoA signaling. J. Cell Biol. 196, 147–162 (2012). 22232705 10.1083/jcb.201102085 PMC3255987

