# Supplementary material for: PCTK3/CDK18 regulates cell migration and adhesion by negatively modulating FAK activity
Source: Sci Rep. 2017 Mar 31;7:45545. doi: 10.1038/srep45545 (PMC5374530; doi:10.1038/srep45545)
Supplement: Supplementary Figures [file srep45545-s1.pdf]

PCTK3/CDK18 regulates cell migration and adhesion by negatively modulating FAK activity

Shinya Matsuda[1], Kohei Kawamoto[1], Kenji Miyamoto[1], Akihiko Tsuji[1,2], and Keizo Yuasa[1,2,*]

[1] *Department of Biological Science and Technology, Tokushima University Graduate School, Minamijosanjima, Tokushima 770-8506, Japan*

[2] *Department of Bioscience and Bioindustry, Tokushima University Graduate School, Minamijosanjima, Tokushima 770-8513, Japan*

Supplementary information:

Supplementary Figure 1

Supplementary Figure 2

Supplementary Figure 3

Supplementary Figure 4

Supplementary Figure 5

Supplementary Figure 6

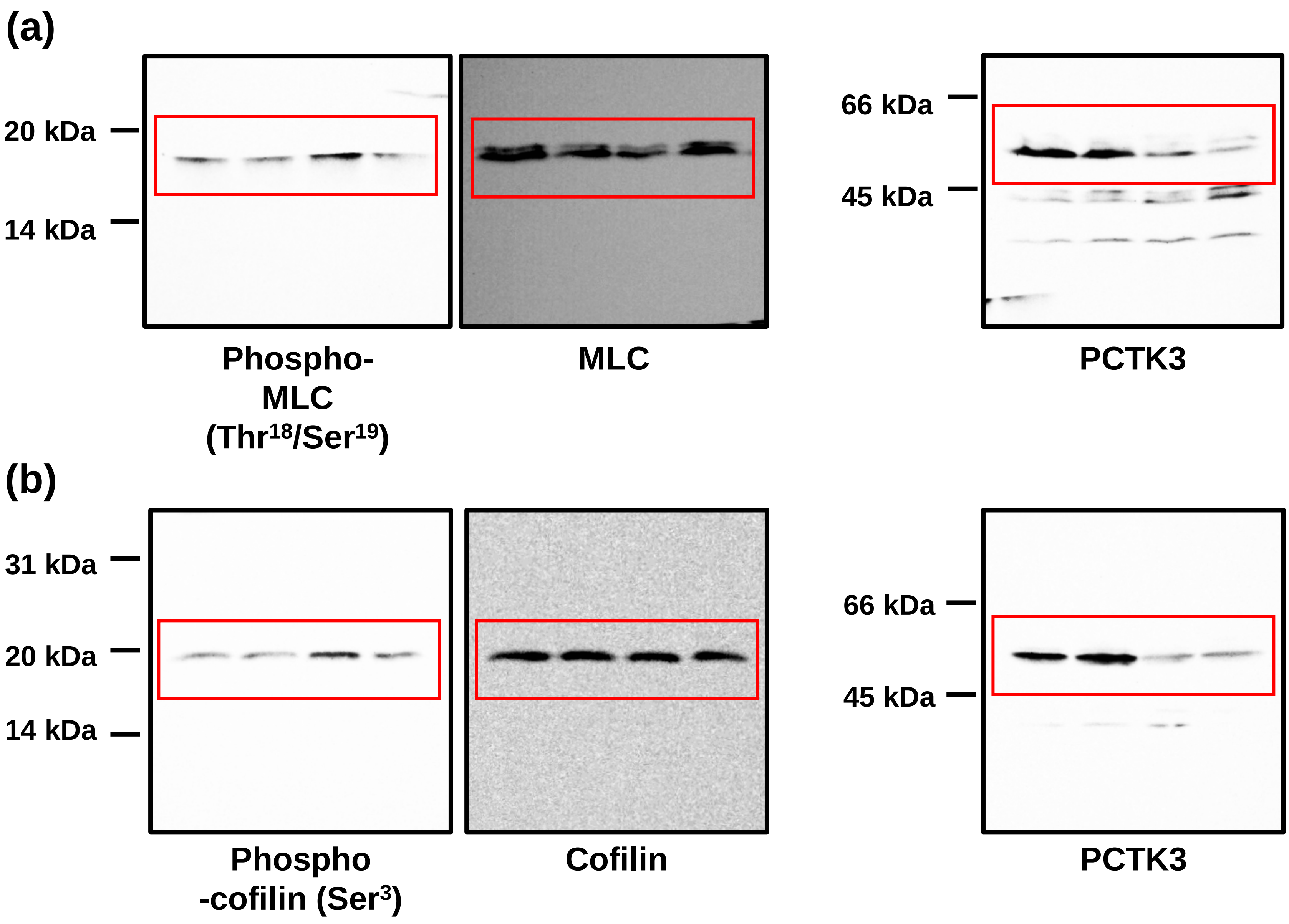

Supplementary Figure 1:
Full-length blots corresponding to Fig. 1. (a) Indicated parts (red box) are shown in Fig. 1e. (b) Indicated parts (red box) are shown in Fig. 1f.

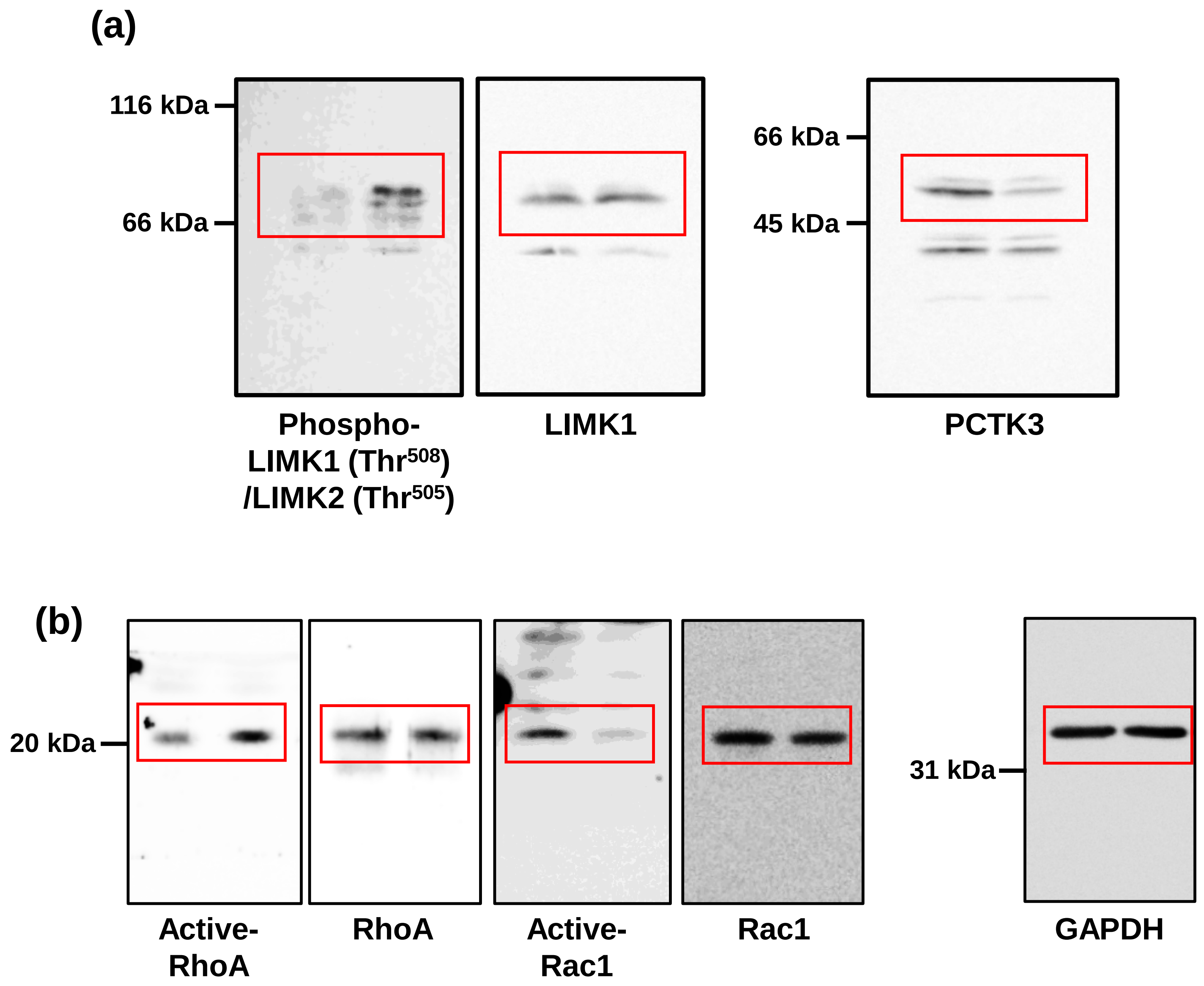

Supplementary Figure 2:
Full-length blots corresponding to Fig. 2. (a) Indicated parts (red box) are shown in Fig. 2a. (b) Indicated parts (red box) are shown in Fig. 2c.

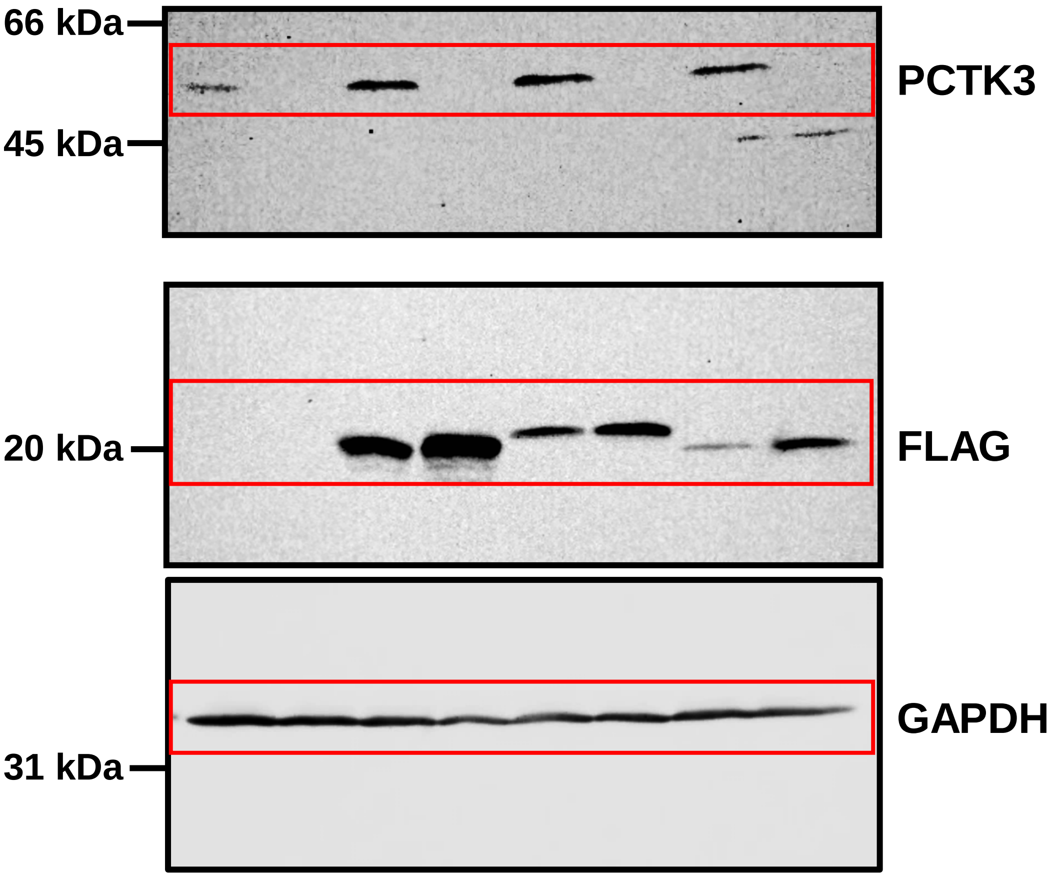

Supplementary Figure 3:
Full-length blots corresponding to Fig. 3. Indicated parts (red box) are shown in Fig. 3b.

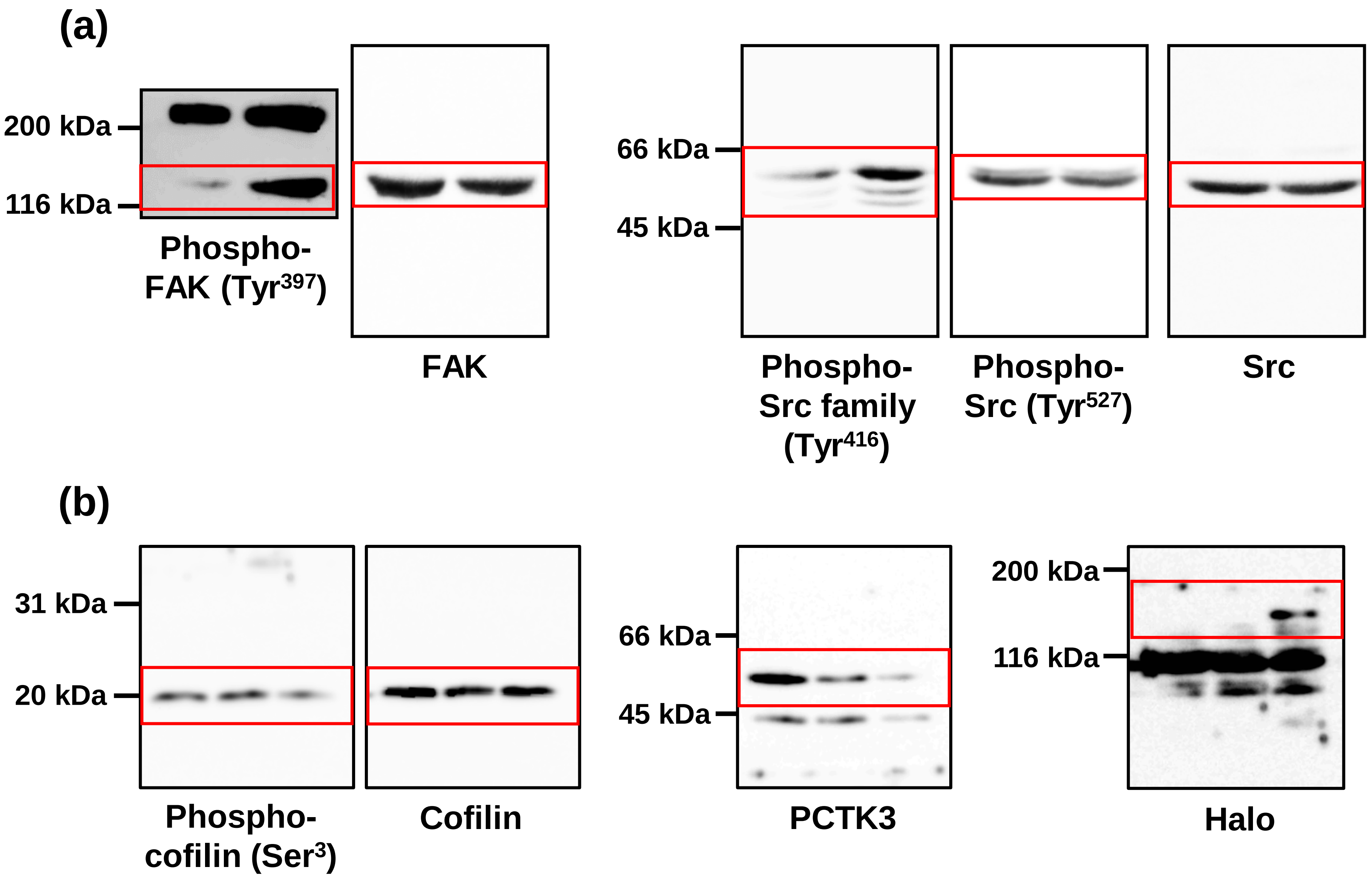

Supplementary Figure 4:
Full-length blots corresponding to Fig. 4. (a) Indicated parts (red box) are shown in Fig. 4a. (b) Indicated parts (red box) are shown in Fig. 4b.

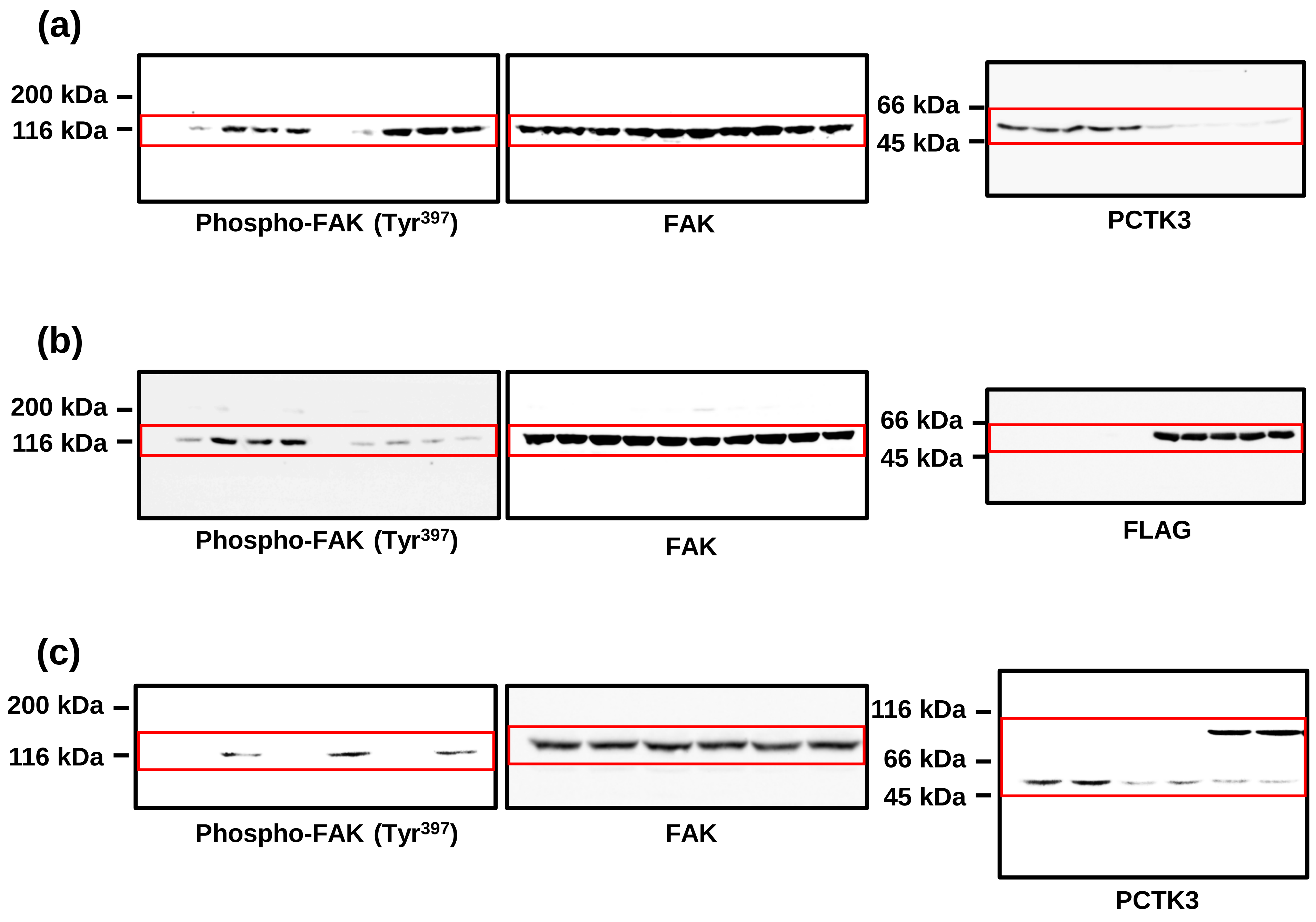

Supplementary Figure 5:
Full-length blots corresponding to Fig. 5. (a) Indicated parts (red box) are shown in Fig. 5a. (b) Indicated parts (red box) are shown in Fig. 5b. (c) Indicated parts (red box) are shown in Fig. 5c.

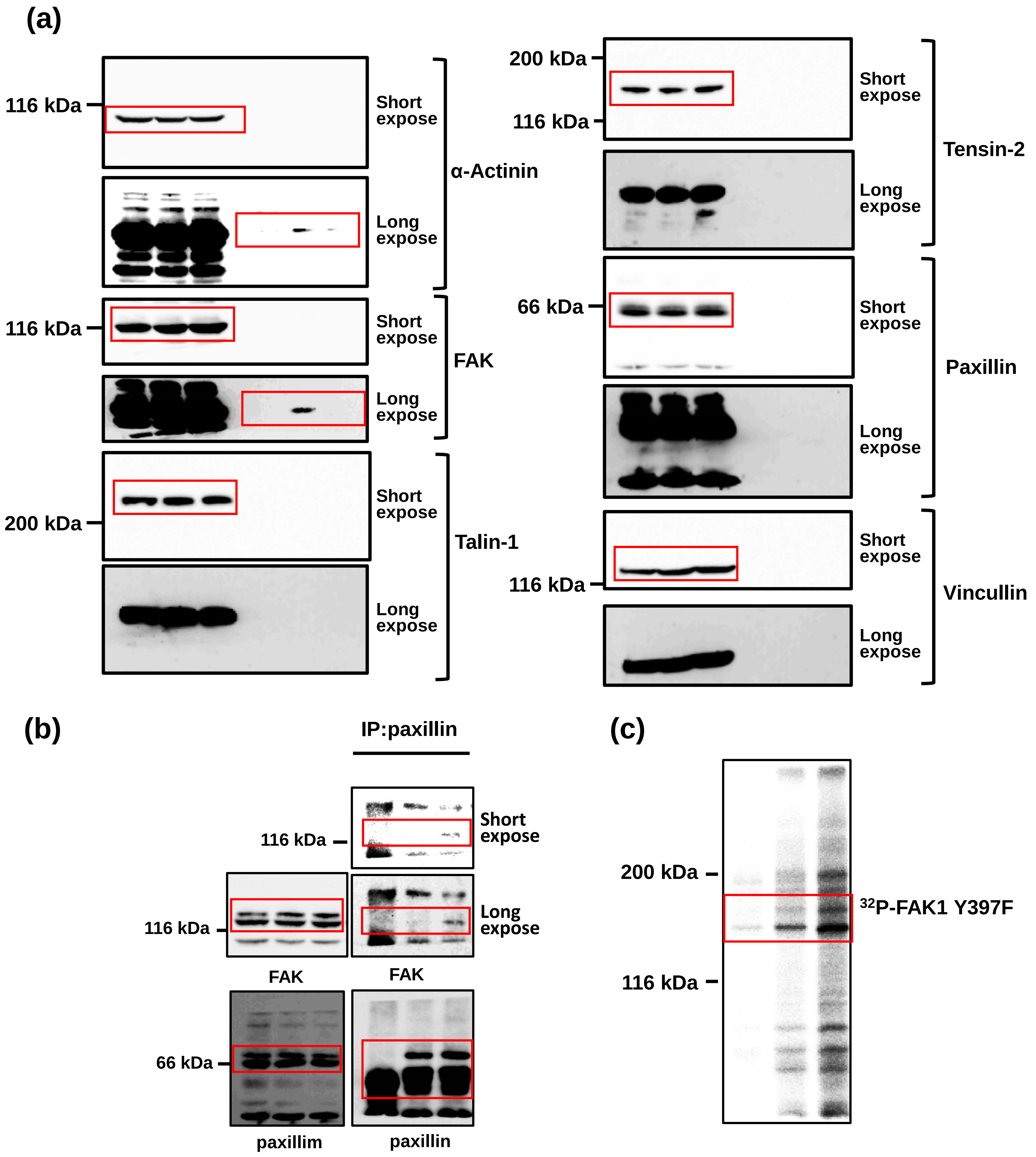

Supplementary Figure 6:
Full-length blots corresponding to Fig. 7. (a) Indicated parts (red box) are shown in Fig. 7a. (b) Indicated parts (red box) are shown in Fig. 7d. (c) Indicated parts (red box) are shown in Fig. 7f.
